# A perspective on the role of physiological stresses in cancer, diabetes and cognitive disease as environmental diseases

**DOI:** 10.3389/fmolb.2023.1274221

**Published:** 2023-11-20

**Authors:** Maranda Esterhuizen, Chang-Beom Park, Young Jun Kim, Tae-Young Kim, Hakwon Yoon, Frederic Andres, Rosalia Rodriguez-Rodriguez, Shihori Tanabe

**Affiliations:** ^1^ Ecosystems and Environment Research Programme, Faculty of Biological and Environmental Sciences, University of Helsinki, Lahti, Finland; ^2^ Environmental Exposure and Toxicology Research Center, Korea Institute Toxicology (KIT), Jinju, Republic of Korea; ^3^ Korean Institute of Science and Technology Europe (KIST Europe), Saarbrücken, Germany; ^4^ School of Earth Sciences and Environmental Engineering, Gwangju Institute of Science and Technology (GIST), Gwangju, Republic of Korea; ^5^ Digital Content and Media Sciences Research Division, National Institute of Informatics, Tokyo, Japan; ^6^ Department of Basic Sciences, Faculty of Medicine and Health Sciences, Universitat Internacional de Catalunya (UIC Barcelona), Barcelona, Spain; ^7^ Centro de Investigación Biomédica en Red de Fisiopatología de la Obesidad y la Nutrición (CIBEROBN), Instituto de Salud Carlos III, Madrid, Spain; ^8^ Division of Risk Assessment, Center for Biological Safety and Research, National Institute of Health Sciences, Kawasaki, Japan

**Keywords:** environmental stressors, reactive oxygen species, environmental disease, human health, cancer, cognitive function, diabetes

## Abstract

With rapid industrialization, urbanization, and climate change, the impact of environmental factors on human health is becoming increasingly evident and understanding the complex mechanisms involved is vital from a healthcare perspective. Nevertheless, the relationship between physiological stress resulting from environmental stressors and environmental disease is complex and not well understood. Chronic exposure to environmental stressors, such as air and water contaminants, pesticides, and toxic metals, has been recognized as a potent elicitor of physiological responses ranging from systemic inflammation to immune system dysregulation causing or progressing environmental diseases. Conversely, physiological stress can exacerbate susceptibility to environmental diseases. Stress-induced alterations in immune function and hormonal balance may impair the ability to detoxify harmful substances and combat pathogens. Additionally, prolonged stress can impact lifestyle choices, leading to harmful behaviors. Understanding the link between physiological stress and environmental disease requires a systematic, multidisciplinary approach. Addressing this complex relationship necessitates the establishment of a global research network. This perspective discusses the intricate interplay between physiological stress and environmental disease, focusing on common environmental diseases, cancer, diabetes, and cognitive degeneration. Furthermore, we highlight the intricate and reciprocal nature of the connection between physiological stress and these environmental diseases giving a perspective on the current state of knowledge as well as identifying where further information is necessary. Recognizing the role of physiological stress in environmental health outcomes will aid in the development of comprehensive strategies to safeguard public health and promote ecological balance.

## 1 Introduction

**FIGURE 1:**
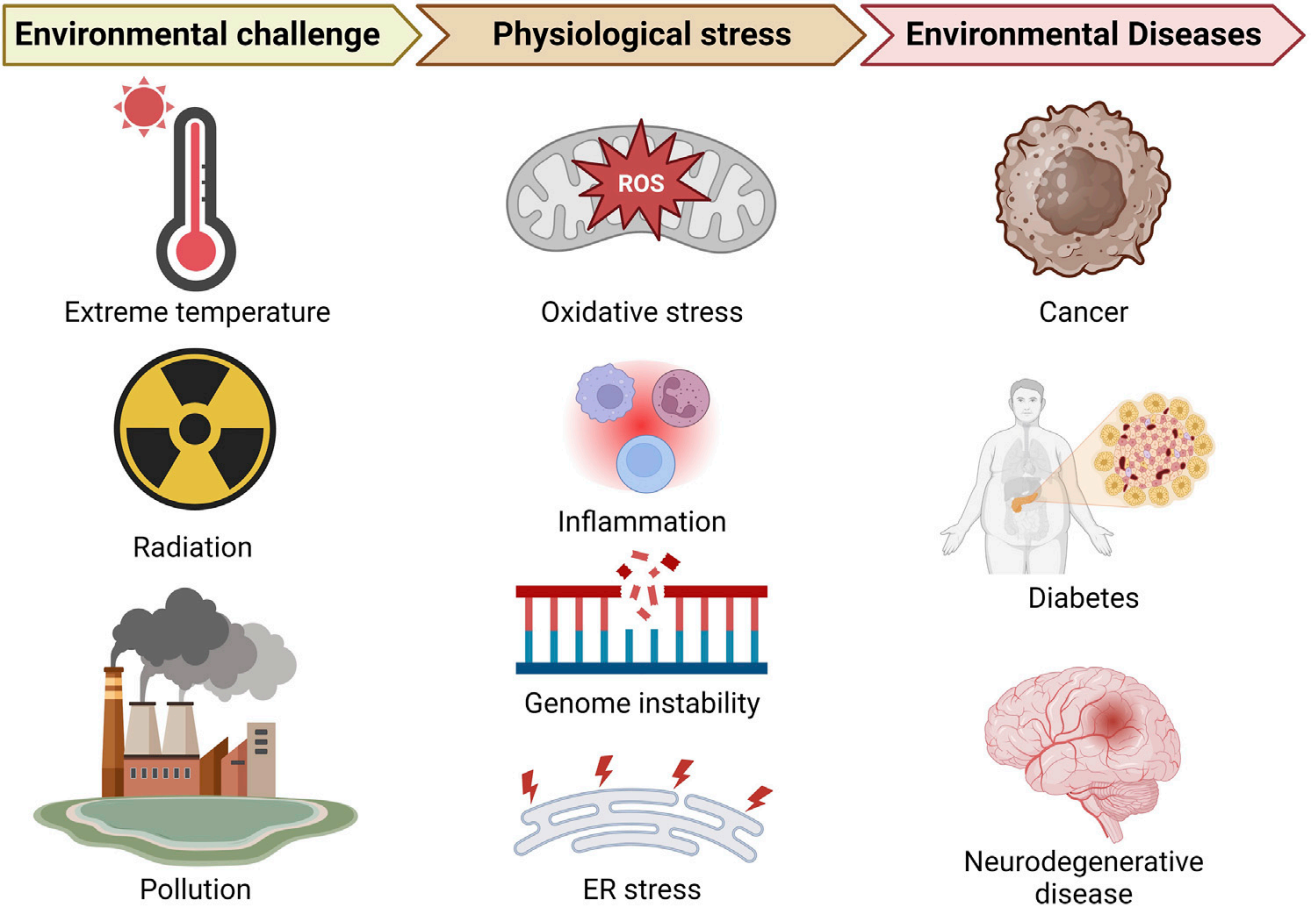
Compact depiction of physiological stress linking selected environmental stressors and the environmental diseases discussed in this perspective (created with the aid of BioRender.com).

Failure of an organism to respond adequately to stimuli, whether originating internally or externally, resulting in the disruption of cellular homeostasis can be classified as stress. Stress may stem from physical, physiological, and psychological sources. Physiological stress primarily occurs when the body faces environmental challenges, which alter normal physiological functionality, which is the focus of this perspective. Environmental stresses encompass a wide range of factors, including exposure to extreme temperatures, radiation, toxins, and pollution, including inadequate access to clean water and sanitation. These stressors can lead to oxidative stress which may result in lipid peroxidation, DNA mutations or damage, and protein oxidation, as well as weaken the immune system, cause inflammation, contributing to environmental diseases (Lovallo, 2005; Thanan et al., 2014) as depicted in Figure 1. For example, Thanan et al. (2014) reviewed the role of oxidative stress stemming from exposure to environmental factors in the development and progression of cancer and neurodegenerative disease. Furthermore, collection of Adverse Outcome Pathways (AOPs) dedicated to understanding the effects of reactive oxygen species (ROS) originating from environmental stressor exposure, related to disease development and progression, has been established with several hundreds of AOPs registered (Tanabe et al., 2022; Tanabe et al., 2023). Thus, the environment is central to human health in terms of disease development and progression.

At a cellular level, responses to stress are mediated by multifaceted interactions, including the nervous, endocrine, and immune systems and are adaptive to counteract acute instances effectively. However, repetitive or prolonged, chronic exposure to stress may cause maladapted responses and impact cellular physiology, resulting in the development and exacerbation of many diseases (Yang et al., 2014; Ketchesin et al., 2017). Furthermore, stress responses primarily mediated by the release of stress hormones like cortisol, can have profound effects on immune, metabolic and cognitive functions, as well as cardiovascular health, and neurological function (Russell and Lightman, 2019). Moreover, a weakened immune system could contribute to susceptibility to infections and diseases (Pedersen et al., 2011).

Individuals with compromised immune systems are more susceptible to developing respiratory issues, allergies, and autoimmune disorders related to environmental stresses (Glaser and Kiecolt-Glaser, 2005). Excessive inflammation has been associated with cardiovascular problems, respiratory disorders, and specific types of cancer (Steptoe et al., 2007; Manisalidis et al., 2020). Environmental pollutants can also induce oxidative stress, accelerating cell damage and increasing the risk of cancer, neurodegenerative disorders, and respiratory conditions (Halliwell and Gutteridge, 2015). Furthermore, hormonal disruption, caused by endocrine disruptors like pesticides and plasticizers, can impact reproductive health and raise the likelihood of hormone-related cancers (Zoeller et al., 2012). Moreover, neurotoxic substances such as heavy metals and organophosphates can impair neuronal development, leading to behavioral disorders and neurodegenerative diseases (Grandjean and Landrigan, 2006).

Many of the toxic effects induced by environmental stressors have been found to be mediated by the regulation or induction of apoptosis and redox signaling (West, 2000; Abdollahi et al., 2004; Assefa et al., 2005; Ryter et al., 2007; Valko et al., 2007), and their deregulation associated to the etiology of many environmental diseases (Fadeel and Orrenius, 2005). Although redox signaling has been primarily linked to activating distinct apoptotic pathways in response to environmental stress, the direct molecular mechanisms involved remain elusive. In other words, although many toxicological studies have investigated cellular responses to environmental stress and the implication of oxidative stress in disease [reviewed by Kruk et al. (2019)], a clear understanding of the mechanistic events related to disease onset and progression is still lacking. The involvement of oxidative stress in the development of selected environmental diseases will be dealt with in more detail in the following sections.

As research towards understanding how environmental risk factors influence the development and progression of environmental diseases will lead to improved public health, understanding the underlying mechanisms in physiological stress related to disease becomes evident. In this perspective article, the current state of knowledge on the implication of physiological stress in environmental disease will be concisely assessed, especially concerning cancer, diabetes, and cognitive diseases, highlighting the knowledge gaps in understanding the mechanisms relating to physiological stress, and particularly oxidative stress, to disease etiology. We highlight existing and identify knowledge gaps that require further investigation to comprehend the underlying mechanisms involved in developing environmental diseases.

## 2 Cancer

Environmental risk factors, such as exposure to carcinogens and pollutants, contribute to the onset and progression of cancer. Petrinović et al. (2023) reviewed inflammation as a link between stress and cancer. Furthermore, physiological stress has been implicated in the activation of tumor progression genes (Zweitzig et al., 2007). By contrast, oxidative stress is considered a driver of cancer dormancy; however, disease recurrence after prolonged dormancy is a significant cause of cancer-associated mortality (Payne, 2022). The balance of the microenvironment is thought to be involved in cancer development (Chipurupalli et al., 2019). Hypoxic microenvironment condition induces adaptive responses such as endoplasmic reticulum (ER) stress response, unfolded protein response, anti-oxidative responses, and autophagy in cancer, which allows the adaption to a stressful microenvironment of cancer (Chipurupalli et al., 2019). Autophagy is a catabolic intracellular nutrient scavenging pathway triggered by nutrient deprivation and stress, which is upregulated in many cancers and in response to cancer therapy to confer treatment resistance (White et al., 2021). Genome instability, which is the consequence of DNA lesions that can result from errors in DNA replication, from the action of genotoxic compounds, including cellular metabolites or from ultraviolet (UV) and ionizing radiation, is a hallmark of cancer (Gaillard et al., 2015). DNA replication stress is a feature present in most cancers, which is induced by oncogenes inducing sustained proliferation and induces other cancer hallmarks, escape from apoptosis and genomic instability (Macheret and Halazonetis, 2015).

A recent review has proposed that ROS contribute to gastric cancer vascularization (Biagioni et al., 2023). Chronic ROS and oxidative stress can consequently suppress the antioxidant system and induce several signaling pathways such as interleukin-6 receptor (IL-6R)/gp130/STAT3 signaling pathway, leading to radiotherapy-resistant gastric cancer (Gu et al., 2018). Despite the already advanced insights into cancer development and progression, the complete mechanisms that link chronic stress to cancer remain incompletely understood. Nevertheless, physiological stresses clearly play central roles in cancer etiology and elucidation of this link is vital for the advancement of cancer therapeutics.

## 3 Diabetes

The acute activation of stress-related neuroendocrine systems contributes to maintain homeostasis; however, chronic and excessive stress can play a decisive role in the onset and progression of metabolic diseases such as type 2 diabetes (Hackett and Steptoe, 2017; Kautzky et al., 2022; Kivimäki et al., 2023). In response to stress, rapid alterations in central and peripheral metabolism and hormone trafficking occur by several biological pathways contributing to diabetes etiology: i) upregulation of the hypothalamic-pituitary-adrenal (HPA) axis with cortisol release, ii) activation of the sympathetic nervous system, and iii) inflammatory processes and oxidative stress. Under chronic stress, release of the corticotropin-releasing hormone from the hypothalamus leads to HPA axis activation, finally target adrenal cortex and promote the release of glucocorticoid cortisol, with relevant functions, many of them related to glucose homeostasis and mobilization of energy stores (Kagias et al., 2012; Kuo et al., 2015; Li and Cummins, 2022). Glucocorticoids stimulate hepatic gluconeogenesis and glycogen depletion, attenuate glucose update in muscle and adipose tissue, and antagonize insulin metabolic actions, promoting hyperglycemia and insulin resistance (Kuo et al., 2015; Li and Cummins, 2022). Cortisol-related stress pathways can also gradually induce visceral fat accumulation and pancreatic β-cell production and secretion of insulin. Activation of the immune system and chronic inflammatory processes is also a crucial mechanism by which stress influences the risk of developing diabetes (Donath and Shoelson, 2011). Chronic hyperglycemia is also leading to cellular disruption with mitochondrial dysfunction, ROS production, ER stress, and alterations of autophagy (Burgos-Morón et al., 2019). Cortisol release in response to stress, increases blood pressure and heart rate via sympathetic system activation (Brotman et al., 2007; Ulrich-Lai and Herman, 2009), thanks to the release of adrenaline, which also induces energy mobilization and the release of pro-inflammatory cytokines and insulin resistance (Emdin et al., 2005; Brotman et al., 2007; Ulrich-Lai and Herman, 2009).

Altogether, chronic changes in several stress-responsive biological systems influence glucose homeostasis and insulin metabolic actions and subsequently increase the risk of diabetes. Interventions based on stress management seem to alleviate stress symptoms and glycemia in patients with type 2 diabetes, but the impact of this intervention on disease progression has not been clearly established yet. Also, whether glucose and insulin levels can be directly disrupted by chronic stress or indirectly affected through these pathways is a matter of debate.

## 4 Cognitive function and neurodegradation

The interplay between environmental risk factors and their impact on cognitive function and neurological disorders has become an increasingly significant area of concern in recent years. While stress is well-documented for its capacity to induce structural alterations in the brain, thereby influencing cognition and memory (Lupien et al., 2009), a broader spectrum of environmental stressors is now being recognized for their potential role in this complex relationship.

Beyond psychological stress, which is known to affect cognitive function, environmental stressors such as extreme temperature conditions (Taylor et al., 2016; Martin et al., 2019) and exposure to pollutants have been found to significantly impact cognitive performance. For example, Kishore et al. (2013) demonstrated that the ingestion of tyrosine can enhance the preservation of cognitive function during passive heat stress, correlated with increased levels of dopamine and epinephrine. Despite this indication of the importance of tyrosine, the underlying mechanism remains elusive.

Furthermore, it is crucial to acknowledge that other environmental stressors have firmly established their negative influence on cognitive health. Air pollution (Lopuszanska and Samardakiewicz, 2020; Gao et al., 2021) and heavy metal exposure (Wang and Matsushita, 2021) have both been associated with cognitive impairments. In a comprehensive review by Singh et al. (2019), it was highlighted that ROS, stemming from exposure to environmental stressors such as ultraviolet and ionizing radiation, as well as chemical pollution, play a central role in neurodegeneration and neurological disorders.

In addition to environmental stressors, inflammation has emerged as a central contributor to cognitive impairment (Leonardo and Fregni, 2023). The central nervous system’s susceptibility to oxidative damage caused by radicals (Cui et al., 2004) emphasizes the significant role of oxidative stress in the onset and progression of neurodegenerative disorders (Angelova and Abramov, 2018; Singh et al., 2019). This sensitivity arises from the abundance of unsaturated fatty acids and oxygen, making lipid peroxidation a critical process (Cobley et al., 2018). Furthermore, an elevated oxidative stress status, coupled with increased apoptosis, has been shown to contribute to the pathogenesis of neurodegeneration (Bhat et al., 2015). Moreover, free radicals have been implicated in the development and progression of cognitive deficits through their disruptive effects on synaptic transmission, mitochondrial function, neuroinflammation, and axonal transport (Cui et al., 2004; Angelova and Abramov, 2018; Cobley et al., 2018).

While it is evident that oxidative stress and inflammation play pivotal roles, the precise cellular and molecular mechanisms by which stress impairs cognitive function remain incompletely understood. Additionally, our knowledge concerning the impact of diverse environmental stressors on cognitive function and neurodegeneration remains limited. It is imperative to comprehend the link between physiological stress and cognitive function to develop interventions aimed at mitigating the adverse effects of stress on the brain, thus optimizing cognitive performance and overall wellbeing.

Significant strides have been taken in understanding how stress affects cognitive function, yet there exist numerous gaps in our understanding that necessitate further research. These gaps encompass the factors of individual variability, such as age, sex, predisposition, resilience, and more, as well as the timing and duration of stress exposure and the interactions with other contributing factors. The relationship between neurological disorders and environmental influences is intricate and multifaceted, requiring ongoing exploration to uncover the intricate mechanisms, variations among individuals, and the potential for interventions to ameliorate the detrimental effects of stress on cognition.

## 5 Discussion

The link between physiological stress and environmental disease is a complex and significant aspect of public health. The One-Health concept acknowledges the interconnectedness of human health, animal health, and environmental health. It underscores the significance of considering the health of humans, animals, and the environment in an integrated manner to achieve optimal health outcomes. Concerning environmental diseases, the One-Health approach recognizes that environmental factors exert a substantial influence on human health (Lippi et al., 2022). This includes understanding how emerging pollutants such as air particulate matter or emerging pollutants contribute to the development or exacerbation of various diseases in both humans and animals (Flies et al., 2019). By adopting this approach, a better understanding of the intricate interactions between the environment and human health can be achieved, thereby enabling the formulation of more effective strategies for preventing and managing environmental diseases.

This perspective aimed to deliver a concise overview of how physiological stresses can significantly impact human health and increase the risk of developing various environmental diseases. In most cases, the link between chronic physiological stress and disease etiology, primarily occurs via causing oxidative stress, disrupting the body’s stress response system, leading to hormonal imbalances, increased inflammation, and impaired immune function. Thus, prolonged physiological stress can contribute to developing environmental diseases such as cardiovascular disorders, gastrointestinal problems, immune system dysfunction, and mental health disorders.

The relationship between environmental risk factors and environmental diseases has not been clearly elucidated to date, particularly pertaining to molecular mechanisms. To accurately determine the increased disease risk resulting from stress responses, further scientific evidence is required regarding the effects of different stressors, their severity, and the duration of stress (acute or chronic) on the physiological and metabolic responses in the body. In particular, the complex nature of chemical toxicants, such as non-linear dose-response relationships and mixture effects, hinders the attainment of consistent results in toxicological studies (Lee, 2012). Therefore, it is essential to establish a systematic pre- and post-management system for effectively addressing environmental disease studies. This system should encompass the categorization of environmental disease occurrence based on exposure to exogenous risk factors, as well as hazard and risk assessment considering exposure scenarios, symptoms, and induction pathways. Moreover, considering the actual environmental conditions characterized by chronic exposure to low concentrations, future research should be discussed revolving around epigenome-based biomarkers, exposomes, and intergenerational effects (Wild, 2005). Furthermore, since environmental diseases arise from chronic exposure to environmental stressors, it is crucial to demonstrate the risk and persistence of these diseases under various conditions such as age, gender, and health status. A noteworthy consideration for future investigation is individual susceptibility to these stresses which can vary based on genetic factors, lifestyle choices, and overall health status (Rea, 2017; Mancinelli et al., 2021). Furthermore, prolonged exposure may not necessarily directly cause environmental diseases but can significantly increase the risk or exacerbate pre-existing conditions (Li et al., 2023). Thus, understanding the underlying mechanism involved in disease development and progression regulated by environmental factors is essential. To this end, the establishment of a global research network through collaborative efforts with specialized research institutes and researchers worldwide is necessary.

By taking a holistic approach to healthcare, addressing both physiological and environmental stresses, healthcare providers can improve their patient’s overall health and quality of life. It emphasizes the importance of early intervention and promoting a healthy lifestyle to mitigate the impact of stressors on health. Understanding the role of physiological stresses in diseases is crucial for healthcare professionals in terms of prevention, diagnosis, and treatment. Understanding and addressing the connection between physiological stress and environmental disease is crucial for formulating effective public health strategies to protect and promote the wellbeing of individuals in an increasingly polluted and stress-inducing world. By identifying and addressing these stresses, healthcare providers can develop strategies to mitigate their impact on health and improve patient outcomes. This may involve lifestyle modifications, stress management techniques, environmental interventions, and targeted medical interventions. Overall, physiological stresses play a complex and multifaceted role in the development and progression of diseases. By recognizing and managing these stresses, healthcare professionals can help individuals sustain optimal health and wellbeing and ultimately, this knowledge will contribute to improved advanced methodologies aimed at mitigating the impact of environmental risk factors on human health.

## Data Availability

The original contributions presented in the study are included in the article/Supplementary Material, further inquiries can be directed to the corresponding author.

## References

[B1] Abdollahi M. Ranjbar A. Shadnia S. Nikfar S. Rezaie A. (2004). Pesticides and oxidative stress: a review. Med. Sci. Monit. 10, RA141–147. 15173684

[B2] Angelova P. R. Abramov A. Y. (2018). Role of mitochondrial ROS in the brain: from physiology to neurodegeneration. FEBS Lett. 592 (5), 692–702. 10.1002/1873-3468.12964 29292494

[B3] Assefa Z. Van Laethem A. Garmyn M. Agostinis P. (2005). Ultraviolet radiation-induced apoptosis in keratinocytes: on the role of cytosolic factors. Biochim. Biophys. Acta 1755, 90–106. 10.1016/j.bbcan.2005.04.001 15964692

[B4] Bhat A. H. Dar K. B. Anees S. Zargar M. A. Masood A. Sofi M. A. (2015). Oxidative stress, mitochondrial dysfunction and neurodegenerative diseases; a mechanistic insight. Biomed. Pharmacother. 74, 101–110. 10.1016/j.biopha.2015.07.025 26349970

[B5] Biagioni A. Peri S. Versienti G. Fiorillo C. Becatti M. Magnelli L. (2023). Gastric cancer vascularization and the contribution of reactive oxygen species. Biomolecules 13 (6), 886. 10.3390/biom13060886 37371466 PMC10296003

[B6] Brotman D. J. Golden S. H. Wittstein I. S. (2007). The cardiovascular toll of stress. Lancet 370, 1089–1100. 10.1016/S0140-6736(07)61305-1 17822755

[B7] Burgos-Morón E. Abad-Jiménez Z. de Marañón A. M. Iannantuoni F. Escribano-López I. López-Domènech S. (2019). Relationship between oxidative stress, ER stress, and inflammation in type 2 diabetes: the battle continues. J. Clin. Med. 8, 1385. 10.3390/jcm8091385 31487953 PMC6780404

[B8] Chipurupalli S. Kannan E. Tergaonkar V. D’Andrea R. Robinson N. (2019). Hypoxia induced ER stress response as an adaptive mechanism in cancer. Int. J. Mol. Sci. 20 (3), 749. 10.3390/ijms20030749 30754624 PMC6387291

[B9] Cobley J. N. Fiorello M. L. Bailey D. M. (2018). 13 reasons why the brain is susceptible to oxidative stress. Redox Biol. 15, 490–503. 10.1016/j.redox.2018.01.008 29413961 PMC5881419

[B10] Cui K. Luo X. Xu K. Murthy M. V. (2004). Role of oxidative stress in neurodegeneration: recent developments in assay methods for oxidative stress and nutraceutical antioxidants. Prog. Neuro-Psychopharmacol. Biol. Psychiatry 28 (5), 771–799. 10.1016/j.pnpbp.2004.05.023 15363603

[B11] Donath M. Y. Shoelson S. E. (2011). Type 2 diabetes as an inflammatory disease. Nat. Rev. Immunol. 11, 98–107. 10.1038/nri2925 21233852

[B12] Emdin C. A. Anderson S. G. Woodward M. Rahimi K. (2005). Usual blood pressure and risk of new-onset diabetes: evidence from 4.1 million adults and a meta-analysis of prospective studies. J. Am. Coll. Cardiol. 66, 1552–1562. 10.1016/J.JACC.2015.07.059 PMC4595710 26429079

[B13] Fadeel B. Orrenius S. (2005). Apoptosis: a basic biological phenomenon with wide-ranging implications in human disease. J. Intern. Med. 258, 479–517. 10.1111/j.1365-2796.2005.01570.x 16313474

[B14] Flies E. J. Mavoa S. Zosky G. R. Mantzioris E. Williams C. Eri R. (2019). Urban-associated diseases: candidate diseases, environmental risk factors, and a path forward. Environ. Int. 133, 105187. 10.1016/j.envint.2019.105187 31648161

[B15] Gaillard H. García-Muse T. Aguilera A. (2015). Replication stress and cancer. Nat. Rev. Cancer. 15, 276–289. 10.1038/nrc3916 25907220

[B16] Gao H. Shi J. Cheng H. Zhang Y. Zhang Y. (2021). The impact of long-and short-term exposure to different ambient air pollutants on cognitive function in China. Environ. Int. 151, 106416. 10.1016/j.envint.2021.106416 33667754

[B17] Glaser R. Kiecolt-Glaser J. K. (2005). Stress-induced immune dysfunction: implications for health. Nat. Rev. Immunol. 5 (3), 243–251. 10.1038/nri1571 15738954

[B18] Grandjean P. Landrigan P. J. (2006). Developmental neurotoxicity of industrial chemicals. Lancet 368 (9553), 2167–2178. 10.1016/S0140-6736(06)69665-7 17174709

[B19] Gu H. Huang T. Shen Y. Liu Y. Zhou F. Jin Y. (2018). Reactive oxygen species-mediated tumor microenvironment transformation: the mechanism of radioresistant gastric cancer. Oxid. Med. Cell 2018, 5801209. 10.1155/2018/5801209 PMC5892229 29770167

[B20] Hackett R. A. Steptoe A. (2017). Type 2 diabetes mellitus and psychological stress-A modifiable risk factor. Nat. Rev. Endocrinol. 13, 547–560. 10.1038/nrendo.2017.64 28664919

[B21] Halliwell B. Gutteridge J. M. C. (2015). Free radicals in biology and medicine. 5th ed. Oxford: Oxford Academic. 10.1093/acprof:oso/9780198717478.001.0001

[B22] Kagias K. Nehammer C. Pocock R. (2012). Neuronal responses to physiological stress. Front. Genet. 3, 222. 10.3389/fgene.2012.00222 23112806 PMC3481051

[B23] Kautzky A. Heneis K. Stengg K. Fröhlich S. Kautzky-Willer A. (2022). Biological and psychological stress correlates are linked to glucose metabolism, obesity, and gender roles in women. Neuroendocrinology 112, 130–142. 10.1159/000514484 33461207 PMC8985024

[B24] Ketchesin K. D. Stinnett G. S. Seasholtz A. F. (2017). Corticotropin-releasing hormone-binding protein and stress: from invertebrates to humans. Stress 20 (5), 449–464. 10.1080/10253890.2017.1322575 28436309 PMC7885796

[B25] Kishore K. Ray K. Anand J. P. Thakur L. Kumar S. Panjwani U. (2013). Tyrosine ameliorates heat induced delay in event related potential P300 and contingent negative variation. Brain Cogn. 83 (3), 324–329. 10.1016/j.bandc.2013.09.005 24141022

[B26] Kivimäki M. Bartolomucci A. Kawachi I. (2023). The multiple roles of life stress in metabolic disorders. Nat. Rev. Endocrinol. 19, 10–27. 10.1038/s41574-022-00746-8 36224493 PMC10817208

[B27] Kruk J. Aboul-Enein H. Y. Kładna A. Bowser J. E. (2019). Oxidative stress in biological systems and its relation with pathophysiological functions: the effect of physical activity on cellular redox homeostasis. Free Radic. Res. 53 (5), 497–521. 10.1080/10715762.2019.1612059 31039624

[B28] Kuo T. McQueen A. Chen T. C. Wang J. C. (2015). Regulation of glucose homeostasis by glucocorticoids. Adv. Exp. Med. Biol. 872, 99–126. 10.1007/978-1-4939-2895-8_5 26215992 PMC6185996

[B29] Lee D. H. (2012). Endocrine disrupting chemicals and environmental diseases. J. Korean Med. Assoc. 55 (3), 243–249. 10.5124/jkma.2012.55.3.243

[B30] Leonardo S. Fregni F. (2023). Association of inflammation and cognition in the elderly: a systematic review and meta-analysis. Front. Aging Neurosci. 15, 1069439. 10.3389/fnagi.2023.1069439 36815174 PMC9939705

[B31] Li A. Toll M. Martino E. Wiesel I. Botha F. Bentley R. (2023). Vulnerability and recovery: long-term mental and physical health trajectories following climate-related disasters. Soc. Sci. Med. 320, 115681. 10.1016/j.socscimed.2023.115681 36731303

[B32] Li J. X. Cummins C. L. (2022). Fresh insights into glucocorticoid-induced diabetes mellitus and new therapeutic directions. Nat. Rev. Endocrinol. 18, 540–557. 10.1038/s41574-022-00683-6 35585199 PMC9116713

[B33] Lippi L. Sire A. D. Folli A. Turco A. Moalli S. Ammendolia A. (2022). Environmental factors in the rehabilitation framework: role of the one health approach to improve the complex management of disability. Int. J. Environ. Res. Public Health. 19, 155186. 10.3390/ijerph192215186 PMC9690359 36429901

[B34] Lopuszanska U. Samardakiewicz M. (2020). The relationship between air pollution and cognitive functions in children and adolescents: a systematic review. Cogn. Behav. Neurol. 33 (3), 157–178. 10.1097/WNN.0000000000000235 32889949

[B35] Lovallo W. R. (2005). Stress & health: biological and psychological interactions. United States: SAGE Publications Inc. 10.4135/9781452233543

[B36] Lupien S. J. McEwen B. S. Gunnar M. R. Heim C. (2009). Effects of stress throughout the lifespan on the brain, behaviour and cognition. Nat. Rev. Neurosci. 10 (6), 434–445. 10.1038/nrn2639 19401723

[B37] Macheret M. Halazonetis T. D. (2015). DNA replication stress as a hallmark of cancer. Annu. Rev. Pathol. 10, 425–448. 10.1146/annurev-pathol-012414-040424 25621662

[B38] Mancinelli R. Checcaglini F. Coscia F. Gigliotti P. Fulle S. Fanò-Illic G. (2021). Biological aspects of selected myokines in skeletal muscle: focus on aging. Int. J. Mol. Sci. 22, 8520. 10.3390/ijms22168520 34445222 PMC8395159

[B39] Manisalidis I. Stavropoulou E. Stavropoulos A. Bezirtzoglou E. (2020). Environmental and health impacts of air pollution: a review. Front. Public Health 8, 14. 10.3389/fpubh.2020.00014 32154200 PMC7044178

[B40] Martin K. McLeod E. Périard J. Rattray B. Keegan R. Pyne D. B. (2019). The impact of environmental stress on cognitive performance: a systematic review. Hum. Factors 61 (8), 1205–1246. 10.1177/0018720819839817 31002273

[B41] Payne K. K. (2022). Cellular stress responses and metabolic reprogramming in cancer progression and dormancy. Semin. Cancer Biol. 78, 45–48. 10.1016/j.semcancer.2021.06.004 34098105 PMC8642459

[B42] Pedersen A. F. Bovbjerg D. H. Zachariae R. (2011). “Stress and susceptibility to infectious disease,” in The handbook of stress science: biology, psychology, and health (Berlin, Germany: Springer), 425–445.

[B43] Petrinović S. V. Milošević M. S. Marković D. Momčilović S. (2023). Interplay between stress and cancer—a focus on inflammation. Front. Physiol. 14, 1119095. 10.3389/fphys.2023.1119095 37020461 PMC10067747

[B44] Rea I. M. (2017). Towards ageing well: use it or lose it: exercise, epigenetics and cognition. Biogerontology 18 (4), 679–691. 10.1007/s10522-017-9719-3 28624982 PMC5514203

[B45] Russell G. Lightman S. (2019). The human stress response. Nat. Rev. Endocrinol. 15, 525–534. 10.1038/s41574-019-0228-0 31249398

[B46] Ryter S. W. Kim H. P. Hoetzel A. Park J. W. Nakahira K. Wang X. (2007). Mechanisms of cell death in oxidative stress. Antioxid. Redox Signal 9, 49–89. 10.1089/ars.2007.9.49 17115887

[B47] Singh A. Kukreti R. Saso L. Kukreti S. (2019). Oxidative stress: a key modulator in neurodegenerative diseases. Molecules 24 (8), 1583. 10.3390/molecules24081583 31013638 PMC6514564

[B48] Steptoe A. Hamer M. Chida Y. (2007). The effects of acute psychological stress on circulating inflammatory factors in humans: a review and meta-analysis. Brain Behav. Immun. 21 (7), 901–912. 10.1016/j.bbi.2007.03.011 17475444

[B49] Tanabe S. Beaton D. Chauhan V. Choi I. Choi J. Clerbaux L.-A. (2023). Report of the 3rd and 4th mystery of reactive oxygen species conference. ALTEX - Altern. animal Exp. 40 (4), 689–693. 10.14573/altex.2307041 37889188

[B50] Tanabe S. O’Brien J. Tollefsen K. E. Kim Y. Chauhan V. Yauk C. (2022). Reactive oxygen species in the adverse outcome pathway framework: toward creation of harmonized consensus key events. Front. Toxicol. 4, 887135. 10.3389/ftox.2022.887135 35875696 PMC9298159

[B51] Taylor L. Watkins S. L. Marshall H. Dascombe B. J. Foster J. (2016). The impact of different environmental conditions on cognitive function: a focused review. Front. Physiol. 6, 372. 10.3389/fphys.2015.00372 26779029 PMC4701920

[B52] Thanan R. Oikawa S. Hiraku Y. Ohnishi S. Ma N. Pinlaor S. (2014). Oxidative stress and its significant roles in neurodegenerative diseases and cancer. Int. J. Mol. Sci. 16 (1), 193–217. 10.3390/ijms16010193 25547488 PMC4307243

[B53] Ulrich-Lai Y. M. Herman J. P. (2009). Neural regulation of endocrine and autonomic stress responses. Nat. Rev. Neurosci. 10, 397–409. 10.1038/nrn2647 19469025 PMC4240627

[B54] Valko M. Leibfritz D. Moncol J. Cronin M. T. Mazur M. Telser J. (2007). Free radicals and antioxidants in normal physiological functions and human disease. Int. J. Biochem. Cell Biol. 39, 44–84. 10.1016/j.biocel.2006.07.001 16978905

[B55] Wang H. Matsushita M. T. (2021). Heavy metals and adult neurogenesis. Curr. Opin. Toxicol. 26, 14–21. 10.1016/j.cotox.2021.03.006 34056147 PMC8153364

[B56] West I. C. (2000). Radicals and oxidative stress in diabetes. Diabet. Med. 17, 171–180. 10.1046/j.1464-5491.2000.00259.x 10784220

[B57] White E. Lattime E. C. Guo J. Y. (2021). Autophagy regulates stress responses, metabolism, and anticancer immunity. Trends Cancer 7 (8), 778–789. 10.1016/j.trecan.2021.05.003 34112622 PMC8295230

[B58] Wild C. (2005). Complementing the genome with an “exposome”: the outstanding challenge of environmental exposure measurement in molecular epidemiology. Cancer Epidemiol. Biomarkers Prev. 14 (8), 1847–1850. 10.1158/1055-9965.EPI-05-0456 16103423

[B59] Yang C. Guo X. Wang G. H. Wang H. L. Liu Z. C. Liu H. (2014). Changes in tau phosphorylation levels in the hippocampus and frontal cortex following chronic stress. Braz. J. Med. Biol. Res. 47, 237–244. 10.1590/1414-431X20133275 24652321 PMC3982945

[B60] Zoeller R. T. Brown T. R. Doan L. L. Gore A. C. Skakkebaek N. E. Soto A. M. (2012). Endocrine-disrupting chemicals and public health protection: a statement of principles from the Endocrine Society. Endocrinology 153 (9), 4097–4110. 10.1210/en.2012-1422 22733974 PMC3423612

[B61] Zweitzig D. R. Smirnov D. A. Connelly M. C. Terstappen L. W. O’Hara S. M. Moran E. (2007). Physiological stress induces the metastasis marker AGR2 in breast cancer cells. Mol. Cell. Biochem. 306, 255–260. 10.1007/s11010-007-9562-y 17694278

